# Prolonged transfer of feces from the lean mice modulates gut microbiota in obese mice

**DOI:** 10.1186/s12986-016-0116-8

**Published:** 2016-08-23

**Authors:** Maria Kulecka, Agnieszka Paziewska, Natalia Zeber-Lubecka, Filip Ambrozkiewicz, Michal Kopczynski, Urszula Kuklinska, Kazimiera Pysniak, Marta Gajewska, Michal Mikula, Jerzy Ostrowski

**Affiliations:** 1 Department of Gastroenterology, Hepatology and Clinical Oncology, Medical Center for Postgraduate Education, Warsaw, Poland; 2 Department of Genetics, Maria Sklodowska-Curie Memorial, Cancer Center and Institute of Oncology, Roentgena 5, 02-781 Warsaw, Poland

**Keywords:** 16S rRNA sequencing, Feces transplantation, Gut microbiota, High fat diet, Obesity

## Abstract

**Background:**

Transplanting a fecal sample from lean, healthy donors to obese recipients has been shown to improve metabolic syndrome symptoms. We therefore examined the gut microbiota in mice after administering a long-term, high-fat diet (HFD) supplemented with feces from lean mice through the fecal-oral route.

**Methods:**

C57BL6/W mice were allowed to adapt to a non-specific pathogen free (SFP) environment for 2 weeks before being divided into three groups of 16 animals. Animals were fed for 28 weeks with a normal diet (ND), HFD or HFD supplemented with feces from ND-fed mice (HFDS). The composition of colonizing bacteria was evaluated in droppings collected under SPF conditions at the beginning of the study and at 12 and 28 weeks using an 16S Metagenomics Kit on Ion PGM sequencer.

**Results:**

HFD and HFDS-fed mice attained (*p* < 0.05) greater body weights by weeks 6 and 5, respectively. HFDS-fed mice gained more weight than HFD-fed mice by week 25. Both species diversity and richness indices increased with time in HFDS mice only.

**Conclusions:**

Prolonged HFD-fed mice supplementation with feces from lean mice altered bacteria species diversity and richness, accelerated the onset of obesity, and caused increased weight gain in the later weeks of the HFD regimen.

**Electronic supplementary material:**

The online version of this article (doi:10.1186/s12986-016-0116-8) contains supplementary material, which is available to authorized users.

## Background

Obesity results from an imbalance between energy intake and energy utilization. Obesity in both humans and animals is associated with decreased intestinal barrier function, gut inflammation and metabolic endotoxemia that can lead to systemic oxidative stress and chronic low-grade inflammation [1, 2]. Although the mouse and human gut metagenomes are similar at the phylum level, they reveal differences on the species level [3]. However, because of the similarity of human and mice gut microbiota at the functional level [4], rodent diet-induced obesity is an accepted model for studying the behavioral and metabolic consequences of overnutrition.

The symbiotic relationship between commensal bacteria and the gut epithelial and lymphoid tissues gives rise to both innate and adaptive immune defenses to pathogens and anoxious antigens, facilitates dietary nutrient and energy harvesting, and enables fermentation of carbohydrates not otherwise digestible by the human host [5]. The composition of the human gut microbiome changes within 24 h of initiating a high-fat (HF)/low-fiber or low-fat/high-fiber diet [6]. The *Bacteroides* enterotype is most prevalent in animals exposed long-term to diets rich in protein and fat, while the *Prevotella* enterotype is most prevalent in animals exposed to diets rich in carbohydrates and deficient of protein [6]. This parallels the microbiome composition observed in European children on a Western diet versus that seen in children in Burkina Faso living on a high-carbohydrate, low protein diet [7].

While in both humans and mice, the relative proportion of fecal *Bacteroidetes* may be lower and that of *Firmicutes* may be higher in obese individuals [2, 8], the meta-analysis has shown that these differences do not represent a consistent feature distinguishing lean from obese human gut microbiota [9]. Obese-prone rats have a gut microbiota that is distinct from that of obese-resistant rats fed the same HF diet [10]. The obese microbiome has a greater capacity to harvest energy; colonization of germ-free mice with microbiota from obese mice results in significantly more total body fat than does colonization with microbiota from lean animals, all else equal [8]. Also, transfer of microbiota from obese-prone, but not obese-resistant rats, to germ-free mice replicates the obese-prone phenotype [10]. Although obesity-related dysbiosis is functionally similar in humans and mice [4], altering the microbiota with probiotics, prebiotics and antibiotics causes weight loss only in mice, not humans [11].

Recent advances in bacterial culture-independent approaches facilitate investigation of the diversity, complexity and between-host variability of normal and disease-modified gut microbial communities. In this study, we examined the modifications of the intestinal microbial community under long-term exposure to a HF diet using 16S ribosomal RNA (rRNA) gene sequencing of DNA [12] extracted from fecal samples. In addition, we compared the gut microbial profiles of obese mice receiving supplementation of feces from lean mice through the fecal-oral route to profiles of control and obese mice over periods of 12 and 28 weeks.

## Methods

### Experimental design

Forty-eight 12-week-old male C57BL6/W mice were transferred to a non-SPF breeding facility and given 2 weeks to acclimate, during which time all animals were fed the standard diet (normal diet [ND]; 10 % of calories from fat) containing 22 % protein and 4.4 % fat (Labofeed H, Morawski, Poland). Animals were then randomly divided into three groups of 16 mice. Two experimental groups were fed a high-fat diet (HFD) containing 22 % protein and 30 % fat (Morawski, Poland), while the control group was fed ND. The diets of mice in one HFD group were supplemented with feces (HFDS) excreted by mice fed ND. Mice are coprophagic and, therefore, five fecal droppings per recipient mouse were added to the cage every week. Each animal was weighed weekly to an accuracy of 0.1 g. Every 4 weeks, each animal was placed into a metabolic cage with full access to feed and water for 24 h. The feces were collected and stored at −80 °C until use. After 28 weeks, mice were weighed and sacrificed. Blood samples were taken immediately afterwards.

### Animals

C57BL6/W mice were born in a specific pathogen free (SPF) core facility at the Cancer Center Institute, Warsaw and maintained under standard conditions of humidity (55 ± 10 %) and temperature (21 ± 2 °C) in climate-controlled rooms under a 12-h light cycle. Animals had unrestricted access to water and food throughout the experiment. Animals were tested for the presence of viruses, bacteria and parasites according to the recommendations of the Federation of European Laboratory Animal Science Associations (FELASA).

### Serum biochemical measures

Levels of serum glucose, cholesterol, alanine aminotransferase (ALT), aspartate aminotransferase (AST) and alkaline phosphatase (ALKP) were determined by spectrometry on a VITROS DT60-II system (modules DT, DTE, DTSC) using ready-to-use slides (Ortho-Clinical Diagnostics, Johnson & Johnson, Raritan, NJ, USA).

### DNA extraction and metagenome sequencing

Approximately 200 mg of fecal droppings were overlaid with 1 mL InhibitEX Buffer and vortexed thoroughly until homogenized. The sample was then heated at 95 °C for 5 min followed by centrifugation at 14,000 rpm for 2 min using a MiniSpin Plus centrifuge (Eppendorf; Hamburg, Germany) to pellet the stool particles. A sample of the supernatant (200 μL) was transferred into a fresh tube, mixed with 15 μL Proteinase K and 200 μL of AL buffer, and incubated at 70 °C for 10 min. Subsequently, 200 μL of ethanol was added to the sample before transferring to a QIAamp spin column to isolate DNA following the QIAamp DNA Stool Kit protocol (Qiagen, Hilden, Germany). The isolated DNA was quantified with a Nanodrop spectrophotometer (Thermo-Fisher; Waltham, MA, USA) and stored in EB buffer at −20 °C until further analysis.

### 16S rRNA sequencing

Sequencing was performed on an Ion Torrent Personal Genome Machine (PGM) platform (Life Technologies; Carlsbad, CA, USA) using the Ion 16S Metagenomics Kit (Life Technologies; Carlsbad, CA, USA), as previously described [12]. Deep sequencing data have been deposited at The European Bioinformatics Institute (EBI) Metagenomics repository under accession number PRJEB13279.

### Bacterial taxonomic identification

Unmapped bam files were converted to fastq using Picard’s SamToFastq. Additional steps of the analysis were performed using Mothur software [13]. Fastq files were converted into the fasta format. For analyses, we only kept sequences that were between 200 and 300 bases in length, had an average base quality of 20 in a sliding window of 50 bases, and had a maximum homopolymer length of 10. Chimeric sequences were identified with the UCHIME algorithm [14] using default parameters and our sequence collection as the reference database. Chimeric sequences were then removed. The remaining 16S rRNA sequences were classified using the Wang method and the SILVA bacterial 16S rRNA database for reference (release 102), at an 80 % bootstrap cut-off (Additional file 1: Table S1). Rarefraction curves on family level were drawn with MEGAN5 software [15]. The taxonomic profile was created using a modified script from STAMP [16]. In determining taxonomic profile, all hypervariable regions were taken into account since, as we previously described, it reflects better the contents of the sample [12].

### Statistical analysis of differences in weight and biochemical parameters

Differences between weights in groups, as well as differences in biochemical parameters, were assessed with Student’s t-tests.

### Data visualization and statistical analyses of taxonomy

Data visualization, statistical analyses and principal component analysis (PCA) were performed using R and the graphics package ggplot2 [17]. Differences in the first two principal components between groups were evaluated with Mann-Whitney U-tests, or, in the case of paired samples, with Mann-Whitney paired U-tests. For statistical analyses on a taxonomical level, the relative abundance of each operational taxonomic unit (OTU) was computed as the number of sequences ascribed to a given OTU divided by the total number of good quality sequences in a sample. OTUs demonstrating essentially constant abundance (IQR < 0) were removed from taxonomic analyses. Differences between groups were evaluated with Mann-Whitney U-tests. Differences in the *Bacteroidetes/Firmicutes* ratio were determined in the same manner. Changes in taxa abundance over time were evaluated with linear mixed-effects models (using R package lme4) [18]. Three models were considered: a null model (abundance ~ 1 + 1|mice), a model with time as the only covariate (abundance ~ 1 + time + 1|mice), and a model including the interaction of diet and time (abundance ~ 1 + time*diet + 1|mice). Models were compared with likelihood ratio tests. All *p*-values were corrected for multiple hypothesis testing using the Benjamini–Hochberg procedure to minimize the false discovery rate (FDR) [19].

### Analysis of diversity

The Chao1 index of species richness and the Simpson index of community diversity were computed in Mothur [13]. Differences between groups at the same time point were assessed with Student’s *t*-test. Differences between time points were assessed with Student’s paired t-test. Presented calculations are for level 5 of SILVA taxonomy.

## Results

**Fig. 1:**
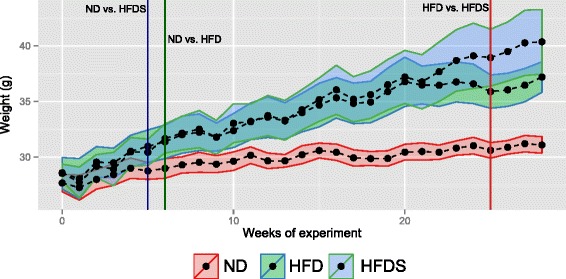
Mean body weights and 95 % confidence intervals of mice in each treatment group throughout the 28 weeks of the experiment. Vertical lines indicate time thresholds from which statistically significant differentiation between groups occurred. ND - normal diet; HFD - high fat diet; HFDS - HFD-fed mice supplemented with feces

As expected, both HFD and HFDS-fed mice gained significantly more weight than ND-fed control mice (Fig. 1). The difference was statistically significant from week 5 onwards for HFDS-fed mice and from week 6 onwards for HFD-fed mice. In addition, the body weight of HFDS-fed mice exceeded that of HFD-fed mice by week 25 (Fig. 1).

Detailed descriptions of the biochemical measures are included in Additional file 1: Table S2. Relative to ND-fed mice, both HFD (*p* = 0.036) and HFDS-fed mice (*p* = 0.0019) developed hypercholesterolemia. Cholesterol level tended to be higher in HFDS than in HFD-fed mice (*p* = 0.056). Mean serum alkaline phosphatase level was lower in HFD-fed mice than in ND-fed mice (*p* = 0.0043). The mean levels of aspartate transaminase, alanine transaminase and glucose did not significantly differ between groups.

### Sequencing results

On average, 8.16 × 10^4^ sequences were generated per library that passed all of the quality filters, and a total of 1.5748596 × 10^7^ good quality sequence reads were generated and assigned to Bacteria or Archaea in the SILVA database. The sequencing depth was deemed to be sufficient with the use of rarefraction curves (Additional file 2: Figure S1). Using SILVA taxonomy, sequences from experimental mice were sorted into 339, 331 and 400 taxa in weeks 0, 14 and 28, respectively. Of these taxa, 57, 73 and 73 in each time point were represented in more than 0.01 % of the reads. Sequences for mice living in the SPF environment sorted into 227 taxa, 68 of which were present in more than 0.01 % of reads. The most abundant phyla found in mice living in the SPF facility were Bacteriodetes, Firmicutes and Proteobacteria (70.6 %, 22.5 %, and 4.8 %) and the proportions of these phyla were similar in experimental animals kept for 2 weeks in non-SPF conditions (week 0: 67.7 %, 28.8 % and 1.5 %). In week 0, Bacteroidetes/Firmicutes ratios in SPF mice (median, 4.94) and non-SPF mice (median, 3.38) did not differ (*p*-value = 0.1195 in Mann-Whitney *U*-test). Between weeks 0 and 12, the proportion of Firmicutes increased significantly and the proportion of Bacteroidetes decreased significantly in all three study groups. Median ratios for ND, HFD and HFDS-fed mice were 1.36, 1.85 and 1.73, respectively (*p*-values: 0.00015, 0.0076 and 0.00042). The ratios did not change further beyond week 12 within or between any of the three groups.

**Fig. 2:**
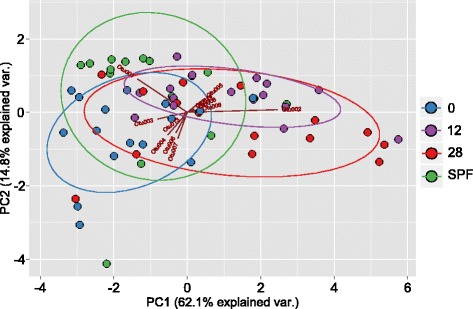
PCA of bacteria taxa abundance in mice kept under specific pathogen free (SFP) conditions at the beginning of the experiment (week 0) and at 12 and 28 weeks after beginning the experiment. Data for weeks 0, 12 and 28 are from mice fed a normal diet (ND). Taxa were identified to SILVA taxonomic level five (family level)

PCA revealed that the most marked differences in bacteriome composition were between mice housed in the SPF environment and those housed for 2 weeks in non-SPF conditions (at week 0). These differences were reflected by variation in the second principal component (*p* = 0.0055). Statistically significant differences in the first principal component were also observed between weeks 0 and 12 (*p* = 9.155e-05 in a paired-test) and weeks 0 and 28 (*p* = 0.007629; Fig. 2). On the other hand, there were no significant differences in the first two principal components between weeks 12 and 28 after the mice had acclimated to the non-SPF environment. Thus, microbial clustering was mostly a product of housing conditions and, to a lesser extent, time.

**Fig. 3:**
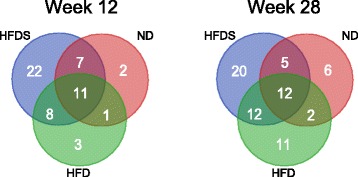
Number of taxa significantly different in abundance between week 0 and weeks 12 and 28 based on paired Mann-Whitney U-tests. Taxa common to more than one experimental group are represented in overlapping sections of the Venn diagrams. ND - normal diet; HFD - high fat diet; HFDS - HFD-fed mice supplemented with feces

**Fig. 4:**
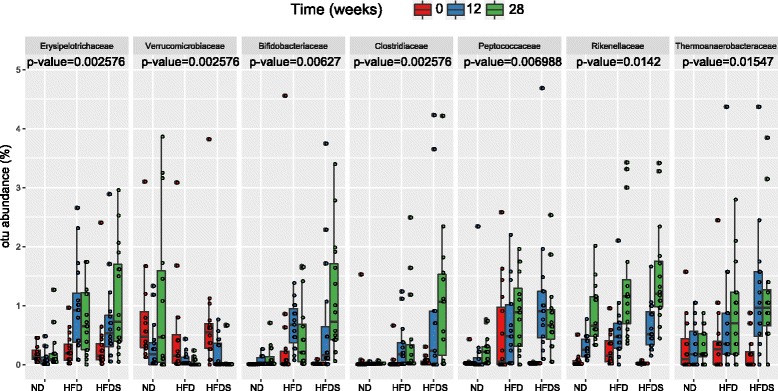
Taxa for which a mixed-effects model including the interaction of time and diet is better than a model including only time. Adjusted p*-value*s from likelihood ratio tests comparing the two models are given under each taxon’s name. ND - normal diet; HFD - high fat diet; HFDS - HFD-fed mice supplemented with feces

To assess variation in the bacteriome over time, we analyzed taxa abundances between weeks 0 and weeks 12 and 28 for each group of mice using paired Mann-Whitney U-tests. We identified 21 (ND), 23 (HFD) and 48 (HFDS) taxa that distinguished mice at week 0 from mice at week 12. Similarly, 25, 37 and 47 taxa distinguished mice at week 28 (Additional file 1: Table S3). Venn diagrams (Fig. 3) illustrate the number of differentially abundant taxa that were common or unique to each group of mice. Of note, while the abundance of seven taxa differed in the gut microbiome of ND-fed mice in week 0 as compared to weeks 12 and 28 (two and five taxa changed in weeks 12 and 28, respectively), the abundance of 13 taxa differed in HFD-fed mice (one taxon changed in both time points, and two and ten taxa changed in weeks 12 and 28, respectively). Supplementation with feces excreted by ND-fed mice further increased the number of differentially abundant taxa to 34; 8, which differentiated time 0 from both 12 and 28 weeks and 14 and 12 taxa which differed in the gut microbiome of HFDS in weeks 12 and 28, respectively (Additional file 1: Table S3). To further explore the changes in bacterial taxa in relation to time and diet, we considered three mixed-effects models. Abundances of 38 (Additional file 1: Table S4) taxa were best fit by a model including time as factor (likelihood ratio test, adjusted *p*-value ≤ 0.05). Abundances of nine taxa were best fit by a model including the interaction of diet and time. Of these taxa, seven were classified to family level (Fig. 4).

To compare experimental groups at each time point, Mann-Whitney U-tests were performed. Differences in taxa abundance between obese mice and lean mice at weeks 12 and 28 were observed (Table 1, Additional file 1: Table S5). In both HFD and HFDS-fed mice, obesity was associated with significant increases in the relative abundances of *Erysipelotrichaceae* (genus: *Turicibacter*), *Clostridiaceae* (genus: *Clostridium*) and *Bifidobacteriaceae* (genus: *Bifidobacterium*) at weeks 12 and 28. Conversely, the abundance of *Verrucomicrobiaceae* (genus: *Akkermansia*) was lower in weeks 12 and 28. In HFD-fed mice, the abundance of *Lactobacillales* (unclassified) was lower in week 12, while *Anaeroplasmataceae* (genus: *Anaeroplasma*) was higher in week 28. Three other taxa were relatively more abundant in HFDS-fed mice: *Peptococcaceae* (genus: *Peptococcus*), *Thermoanaerobacteraceae* (genus: *Thermacetogenium*) and *Peptostreptococcaceae*. Of these families, only *Peptostreptococcaceae* and *Anaeroplasmataceae* abundances were not better fit by the time/diet interaction model than the model that included only time.

**Table 1:** Taxa exhibiting differential abundances (relative to control mice; Mann-Whitney *U*-test) at 12 and 28 weeks of the experiment in high fat diet (HFD) fed mice and HFD-fed mice supplemented with feces (HFDS)

Taxon	HFD		HFDS	
	weeks		weeks	
	12	28	12	28
*Erysipelotrichaceae (Turicibacter)*	up	up	up	up
*Clostridiaceae (Clostridium)*	up	up		up
*Verrucomicrobiaceae (Akkermansia)*		down		down
*Bifidobacteriaceae (Bifidobacterium)*	up			up
*Lactobacillales* (unclassified)	down			
*Anaeroplasmataceae (Anaeroplasma)*		up		
*Peptococcaceae (Peptococcus)*			up	up
*Thermoanaerobacteraceae (Thermacetogenium)*			up	up
*Peptostreptococcaceae*				up

**Fig. 5:**
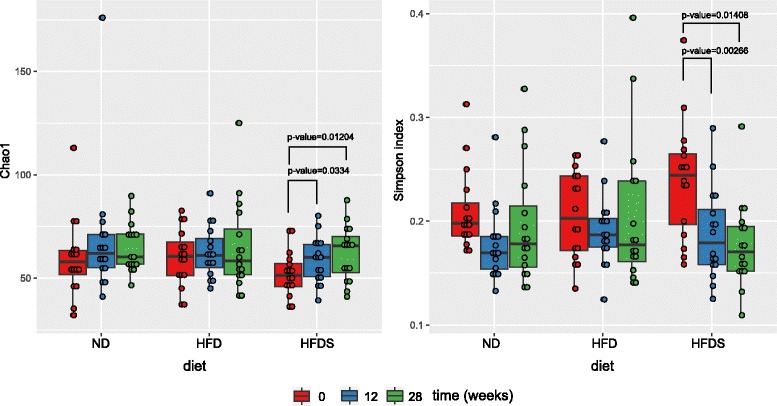
Simpson and Chao indices in weeks 0, 12 and 28 of the experiment for mice fed a normal diet (ND), a high-fat diet (HFD) or HFD supplemented with feces excreted by ND-fed mice (HFDS). The presented *p*-values are from paired t-tests

Compared to week 0, we did not find statistically significant differences in species diversity (as measured by Simpson’s index) or in species richness (as measured by Chao1) between ND and HFD-fed mice in weeks 12 or 28. By contrast, we observed a progressive, statistically significant increase in both species diversity and richness in HFDS-fed mice at both time points (Fig. 5).

## Discussion

While a normal gut microbiota is recognized to contribute to human health, its disruption (dysbiosis) because of environmental exposures leads to numerous disorders, including obesity and obesity-linked co-morbidities [6]. The relatively stable composition of the gut microbiome is modulated by many factors, including diet, sanitation, antibiotics and age [20]. Most alterations of the microbiome’s composition are reversible [21]. In animals, but not humans, altering the microbiota can modulate body weight. The transfer of microbiota from obese animals to germ-free and lean animals results in obesity, while the opposite is observed after introducing microbiota from lean animals to obese animals [11]. However, the lack of intestinal microbiota may not protect mice from diet-induced obesity [22]. Furthermore, although obesity has been associated with phylum-level changes in microbiota composition, reduced bacterial diversity and altered representation of bacterial genes and metabolic pathways, another study reported a lack of a correlation with the proportion of the phyla that were energy harvesters [23] The microbiota may adapt to diet over time, representing a consequence rather than a cause of obesity [8, 11]. Thus, the role of the gut microbiome in the control of body weight and energy homeostasis needs to be further studied.

In line with previous results [4], we found that the mouse gut microbiome was comprised primarily of Bacteriodetes (68.5 %), Firmicutes (27.8 %) and Proteobacteria (1.7 %). These are also the most abundant phyla in the human gut microbiome. Although species diversity and richness were not affected by obesity, the abundances of six families (*Erysipelotrichaceae, Clostridiaceae, Verrucomicrobiaceae, Bifidobacteriaceae, Lactobacillales* and *Anaeroplasmataceae*) were substantially different between lean and obese mice. Among these bacterial taxa, *Erysipelotrichaceae* and *Clostridiaceae* were especially enriched in HFD and HFDS-fed mice, while the abundance of *Verrucomicrobiaceae* was reduced in both groups of obese mice. All of these obesity-induced modifications of the gut microbial profile have been described previously.

Obesity is associated with changes in the relative abundance of the two dominant bacterial divisions, the Bacteroidetes and the Firmicutes [4, 24]. While some studies describe an increased ratio of Firmicutes to Bacteroidetes, other studies show none or even opposite trends [9]. The proportion of gut *Firmicutes* increased under HFD mainly because of the proliferation of the immunogenic bacterial family *Erysipelotrichaceae* [22]. A relatively higher abundance of *Erysipelotrichaceae* has been observed in both obese humans and animals [22, 25], while a lower abundance has been associated with reductions in mice liver injury and intestinal inflammation [26]. In Crohn’s disease patients on low-fat enteral nutrition therapy, the abundance of *Erysipelotrichaceae* is decreased [27]. *Erysipelotrichaceae* abundance has also been correlated with host cholesterol metabolites [28].

*Akkermansia muciniphila* produces a variety of fermentation products that may serve as energy sources for other bacteria and the host [29]. In agreement with earlier studies conducted in genetically and diet-induced obese mice [30], the abundance of *Verrucomicrobiaceae* (including *A. muciniphila*, a mucin-degrading bacterium) was significantly decreased in both obese groups of mice. In humans, *A. muciniphila* was proposed to be a contributor to the maintenance of gut health and glucose homoeostasis, and it has been associated with a healthier metabolic status and better clinical outcomes after calorie restriction in obese adults [31]. Consistent with these observations, treatment of obese mice with *A. muciniphila* reversed weight gain, metabolic endotoxemia and insulin resistance [30].

Fecal transplantation in animals has improved obesity, inflammation, insulin resistance and diabetes [11]. While fecal microbiota transplant is an effective therapy for recurrent *Clostridium difficile* infection in humans (CDI) [32], only one short study of 18 obese men with metabolic syndrome revealed a beneficial effect of the infusion of microbiota from lean donors [33]. On the other hand, development of new-onset obesity was recently reported in a woman treated for CDI with a transplant of feces from a healthy but overweight donor [34].

Mice are normally coprophagic and when lean and obese mice were housed in the same cage, the obese mice were protected from further weight gain by consuming microbiota from lean mice. However, the opposite effect, weight gain in lean mice eating feces from obese mice, was not observed [11]. One hypothesis attributes this observation to differences between lean and obese mice in microbiome diversity that allow the transplantation of the lean microbiome to obese mice, but not vice versa [11]. However, while increased α-diversity and decreased β-diversity after exposure to HFD have been reported [4], we did not observe significant HFD-related changes in species diversity (as measured by Simpson’s index) or in species richness (as measured by Chao1). By contrast, we observed a progressive, statistically significant increase in the both species diversity and richness in HFDS-fed mice (Fig. 5). Specifically, the abundance of 2 (ND), 3 (HFD) and 22 (HFDS) unique taxa differed significantly (adjusted *p*-values ≤ 0.05) between weeks 0 and 12, while 6, 11 and 20 taxa differed by week 28. Prolonged (lasting for 28 weeks) transferring of the gut microbiome from lean mice to HFD-fed mice through the fecal-oral route not only altered species diversity and richness, but also accelerated the onset of obesity. HFDS-fed mice gained more body weight in the last 4 weeks of feeding. Finally, hypercholesterolemia also tended to be more prevalent in HFDS-fed mice.

Abundances of three taxa (*Peptococcaceae, Thermoanaerobacteraceae* and *Peptostreptococcaceae*) appeared to increase significantly with fecal transplants from lean mice to HFD-induced obese mice (Table 1). A high-calorie diet has been associated with an increase in *Peptostreptococcaceae* [35], while an anti-obesity effect of vancomycin treatment in mice on HFD decreased the relative abundances of *Peptostrepococcaceae* and *Peptococcaceae*, both members of the phylum Firmicutes [36]. In addition, dietary intervention with a β-glucan–producing, probiotic lactobacilli strain lowered the proportional abundance of *Peptococcaceae* as compared with the placebo group [37]. Conversely, cellulose supplementation in mice with dextran sulfate sodium (DSS)-induced colitis increased the abundance of *Peptostreptococcaceae* [38]. To date, no association between obesity and the relative abundance of *Thermoanaerobacteraceae* has been described.

As expected, housing conditions markedly influenced the composition of the microbiota in mice (this study and reviewed in [39]) and intestinal barrier integrity in mice fed a HFD [40]. Future studies should consider environmental conditions to be one of the most important factors affecting the composition of the mouse gut microbiome.

## Conclusions

In contrast to previous reports indicating that obesity-related changes of the gut microbiota take place at the phylum level [41], we found rather discrete obesity-related alterations of the mouse microbiome. Furthermore, our data demonstrate that, although transferring feces from lean to HFD-induced obese mice modified the composition of the gut microbiota, this was associated with weight gain instead of the expected weight reduction. These results suggest that there is an unknown compensatory effect that may upend the rationale for treating obesity through microbiota replacement. There is a critical need to search for specific gut microbiota compositions that could be used as therapeutic microbiota transplants.

## Additional files

Additional file 1: Tables S1–S5.
**Table S1. ** Taxonomic Assignments Table. Full taxonomic assignments in SILVA Taxonomy, as classified by Mothur. Taxa are given with bootstrap values for each taxonomic level. Taxa are classified to family, unless there was only one classifiable genus (or lower level) present in the family. **Table S2.** Serum biochemical parameters at the end of the experiment. Normal diet (ND), high-fat diet (HFD), HFD supplemented with feces of ND-fed mice (HFDS). **Table S3.** Differential taxa abundances between weeks 0 and 12 and weeks 0 and 28, tested with paired Mann-Whitney U-tests for each experimental group. Taxon = taxon denotation (full taxonomic assignments in SILVA taxonomy are presented in Additional file 1: Table S1); Mann-Whitney U statistic = statistic from paired Mann-Whitney U-tests; *p*Value = *p*-value from paired Mann-Whitney *U*-test; qValue = *p*-value after FDR correction. **Table S4.** Results of model comparison for taxa. Taxon = taxon denotation (full taxonomic assignments in SILVA taxonomy are presented in Additional file 1: Table S1); *p*Value time and *p*Value diet = *p*-values for comparisons between null models, models including time and models including time and diet, respectively; qValue time and diet = *p*-values after FDR correction. **Table S5.** Differential taxa abundances at 12 and 28 weeks. Taxon = bacteria taxon in SILVA taxonomy, genera (if applicable) are given in parentheses; week = week of experiment; comparison = groups compared; mean 1 group = mean abundance of taxon in first group in comparison field; mean 2 group = mean abundance of taxon in second group; *p*Value = *p*-value of Mann-Whitney U-tests; qValue = *p*-value after FDR correction. Taxa that were differentiated in two groups at both time points are highlighted in bold text. (XLSX 60 kb) Additional file 2: Figure S1. Rarefraction curves from all samples on family level drawn with MEGAN5 software. (PDF 4369 kb)
