# Supplementary figures and images for: Prolonged transfer of feces from the lean mice modulates gut microbiota in obese mice
Source: Nutr Metab (Lond). 2016 Aug 23;13(1):57. doi: 10.1186/s12986-016-0116-8 (PMC4995824; doi:10.1186/s12986-016-0116-8)

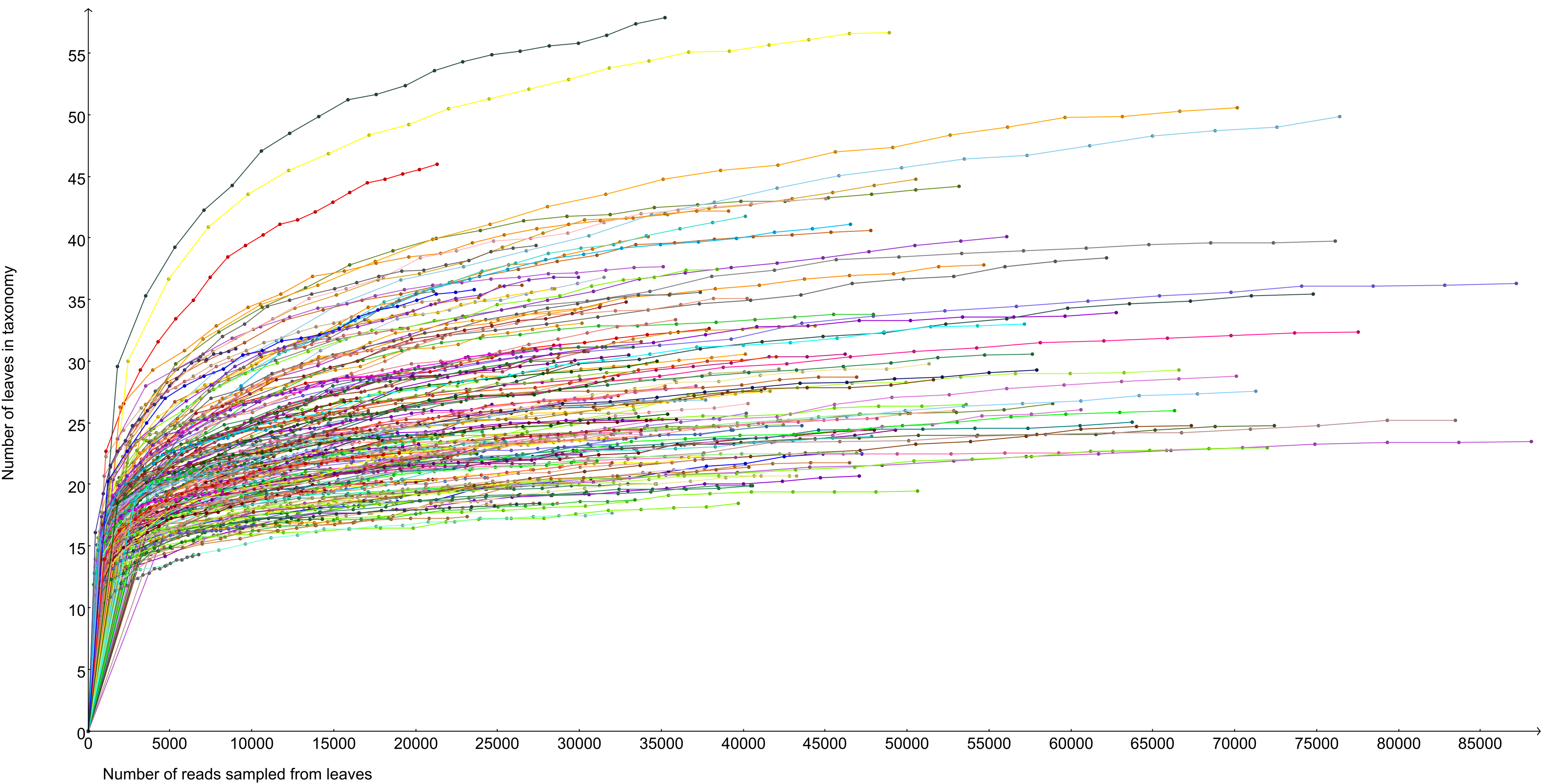

Supplement: Additional file 2: Figure S1. — Rarefraction curves from all samples on family level drawn with MEGAN5 software. (PDF 4369 kb) [file 12986_2016_116_MOESM2_ESM.pdf]
